# Questionnaire-Based Maladaptive Decision-Coping Patterns Involved in Binge Eating Among 1013 College Students

**DOI:** 10.3389/fpsyg.2018.00609

**Published:** 2018-04-26

**Authors:** Wan-Sen Yan, Ran-Ran Zhang, Yan Lan, Zhi-Ming Li, Yong-Hui Li

**Affiliations:** ^1^Department of Psychology, School of Medical Humanitarians, Guizhou Medical University, Guiyang, China; ^2^Key Laboratory of Mental Health, Institute of Psychology, Chinese Academy of Sciences, Beijing, China

**Keywords:** binge eating, decision making, reward discounting, personality, young adults

## Abstract

Binge Eating Disorder (BED), considered a public health problem because of its impact on psychiatric, physical, and social functioning, merits much attention given its elevation to an independent diagnosis in the Diagnostic and Statistical Manual of Mental Disorders, Fifth Edition (DSM-5). Similar with substance use disorders, some neuropsychological and personality constructs are potentially implicated in the onset and development of BED, in which poor decision-making has been suggested to facilitate overeating and BED. The objective of this study was to investigate the associations between decision-coping patterns, monetary decision-making, and binge-eating behavior in young adults. A sample of 1013 college students, equally divided into binge-eating and non-binge-eating groups according to the scores on the Binge Eating Scale (BES), were administered multiple measures of decision-making including the Melbourne Decision-Making Questionnaire (MDMQ), the Delay-discounting Test (DDT), and the Probability Discounting Test (PDT). Compared with the non-binge-eating group, the binge-eating group displayed elevated scores on maladaptive decision-making patterns including Procrastination, Buck-passing, and Hypervigilance. Logistic regression model revealed that only Procrastination positively predicted binge eating. These findings suggest that different dimensions of decision-making may be distinctly linked to binge eating among young adults, with Procrastination putatively identified as a risk trait in the development of overeating behavior, which might promote a better understanding of this disorder.

## Introduction

Compulsive overeating, formally named binge eating disorder (BED) as an independent diagnosis in the Diagnostic and Statistical Manual of Mental Disorders, Fifth Edition (i.e., DSM-5; American Psychiatric Association, 2013), is characterized by consuming large amounts of (mostly highly palatable) food with an overwhelming desire and the associated sense of loss of control (Peat et al., 2017). The lifetime prevalence estimates of DSM-IV BED among adults were 2.8% (3.5% for women and 2.0% for men) in the United States (Hudson et al., 2007) and 1.9% (2.6% for women and 1.1% for men) across 14 WHO World Mental Health (WMH) countries (Kessler et al., 2013). BED is considered a public health problem because it is associated with significant psychiatric and medical complications (Mitchell and Crow, 2010) as well as an increased risk for weight gain and obesity (Stice et al., 2002).

Binge eating disorder has multiple clinical similarities with substance use disorders in some cases, such as the intense cravings for certain foods and a feeling of loss of control even in the face of negative consequences (Davis and Carter, 2009). Besides, BED and addictive disorders share a number of proposed mechanisms including impulsivity and a hyper-reward response to relevant cues (Schulte et al., 2016). There is also substantial overlap between BED and food addiction (Davis, 2017), though the latter topic remains controversial. Akin to addictive disorders, poor decision-making might facilitate overeating. It has been suggested that addictive behaviors are associated with complex cognitive and emotional deficits in the process of decision-making (Redish et al., 2008), thus it is important to acknowledge the possible pathways of decision-making in the onset and development of binge-eating behavior.

Decision making is a complex process involving different choices (e.g., everyday food choices among individuals with BED). There are various decision-making measurements in cognitive neuroscience and behavioral economics. Laboratory cognitive tasks such as the Iowa Gambling Task (IGT; Bechara et al., 1994) mainly test the decision-making abilities of subjects in ambiguity conditions. The Delay-discounting Test (DDT; Kirby et al., 1999) and the Probability Discounting Test (PDT; Madden et al., 2009) primarily measure the individual choice propensity in risk conditions. There are also some self-report personality questionnaires, such as the Melbourne Decision-Making Questionnaire (MDMQ; Mann et al., 1997). The MDMQ characterizes competent decision-making or vigilance as an adaptive appraisal prior to decision-making, while avoiding decisions (i.e., procrastination and buck-passing) or quickly making a choice to escape the uncomfortable feeling of decision making (i.e., hypervigilance) are considered maladaptive traits (Gorodetzky et al., 2011).

Despite scarce evidence, several previous studies have investigated the cognitive profile including decision-making in patients with BED. In one study (Svaldi et al., 2010), women with obesity and BED displayed impaired decision-making in comparison with overweight women without BED on the Game of Dice Task (GDT; Brand et al., 2005), which assesses decision-making under risk with explicit rules for gains and losses. In another study, though female individuals with obesity and BED had worse decision-making performance on the IGT and a Delay Discounting measure compared with normal-weight females, these group differences vanished when education level was taken into account, and there were no task differences between overweight women with and without BED (Davis et al., 2010). Nevertheless, women patients with BED displayed decision-making deficits on the IGT compared to healthy women, which seemed comparable with the poor decision-making performance of women with obesity in this study (Danner et al., 2012). More recently, Aloi et al. (2015) compared decision-making, central coherence and set-shifting functions between women BED patients, Anorexia Nervosa (AN) patients, and healthy controls with a large sample. The results revealed that both BED and AN patients had significantly lower IGT scores compared with healthy controls, showing impaired capacities to advantageously utilize feedback processing in decision making. A functional neuroimaging study of reward-based decision-making in BED has further observed impaired behavioral adaptation in BED patients compared to healthy individuals, accompanied by diminished activation in the anterior insula/ventro-lateral prefrontal cortex (related to exploratory decisions) and reduced representation of ventro-medial prefrontal learning signatures (associated with successful decision-making) (Reiter et al., 2017). Albeit these limited studies and discrepant results, targeting a more precise profile of decision-making in BED may provide a better understanding of the pathogenesis.

The present study thus employed the MDMQ that describes individual decision-coping patterns in daily life at the personality-trait level, as well as the DDT and the PDT that evaluate personal preference in hypothetical money reward choices at the behavioral level, aiming to further investigate the relationships between different dimensions of decision-making and BED with a relatively large sample of general population. It was hypothesized that individuals with binge eating would be more likely to show a steeper delay discounting and a trend toward decreased discounting of probabilistic rewards than individuals without binge eating, and exhibit more maladaptive decision-making patterns on the MDMQ.

## Materials and Methods

### Participants and Procedure

The data were collected in November 2016. Participants included 1050 young adult students, recruited from 12 randomly selected 1st-year courses at a local university in Guiyang, China. All of them were invited to carry out a battery of self-report questionnaires including the demographic information in a 45-min psychology class. The inclusion criteria were: (1) ≥18 years of age, and (2) willingness to participate in this study. The exclusion criteria included current/past major psychiatric disorders (e.g., schizophrenia, major depressive disorder), a history of brain injury/trauma, current/past neurological diseases or mental disorders, and current/past use of psychoactive drugs (e.g., cocaine, heroin, and methamphetamine) by self-report. Thirty-seven students were excluded according to one or more of these exclusion criteria. Finally, 1013 students (average age = 18.85, ranging from 18 to 24 years) were included in data analyses. All subjects provided written informed consent and were compensated with a gift equal to RMB ¥50. This study was approved by the Human Research Ethics Committee at the Guizhou Medical University. Our proposed recruitment process, study design, and plans to compensate participants were consistent with the Declaration of Helsinki.

### Binge Eating Classification

Binge-eating status was classified by employing the Binge Eating Scale (BES; Gormally et al., 1982), which is used to identify individuals with binge-eating behavior, to evaluate binge-eating severity and also as a parameter of treatment outcome (Freitas et al., 2006). BES is a 16-item self-report questionnaire, with a total score ranging from 0 to 46. Subjects scoring 17 and less on the BES are considered individuals without binge eating, and those with a score ≥18 are considered individuals with binge eating (Marcus et al., 1988; Greeno et al., 1995; Ricca et al., 2000). In our study, the Chinese version of the BES (He et al., 2014; Wu et al., 2015) was used. Cronbach’s α for the scale was 0.77. There were 85 persons in the binge-eating group (*M*_*BES*_ = 21.49) and 928 persons in the non-binge-eating group (*M*_*BES*_ = 7.28) according to BES scores.

### Decision-Coping Patterns

The MDMQ (Mann et al., 1997) was used to assess adaptive and maladaptive decision-making traits in the binge-eating and non-binge-eating groups. MDMQ is a 22-item self-report inventory measuring four major coping patterns based on the conflict theory of decision-making (Janis and Mann, 1977). It consists of four subscales (i.e., vigilance, procrastination, buck-passing, and hypervigilance) scoring on a three-point scale: “not true for me” = 0, “sometimes true” = 1, “true for me” = 2. According to Mann et al. (1997), the adaptive or competent decision-making pattern might be characterized by higher scores on the *vigilance* scale (e.g., I like to consider all of the alternatives when making decisions), and avoidant patterns could be indicated by higher scores on the *procrastination* scale (e.g., Even after I have made a decision I delay acting upon it) and *buck-passing* scale (e.g., I prefer to leave decisions to others), while impulsivity and defective decision-making might be indicated by higher *hypervigilance* scores (e.g., I cannot think straight if I have to make a decision in a hurry). In the current study, the Chinese version of the MDMQ (Mann et al., 1998; Zhou et al., 2014) was adopted, and Cronbach’s α for the four subscales was 0.73 (vigilance), 0.66 (procrastination), 0.75 (buck-passing), and 0.63 (hypervigilance), respectively.

### Reward Discounting Measures

The DDT (Kirby et al., 1999) and PDT (Madden et al., 2009) were employed to assess the discounting degree of delayed or probabilistic rewards in the context of monetary decision-making. The DDT is a 27-item choice questionnaire between immediate but smaller and delayed but larger monetary rewards. Delay discounting implies a trend that subjects prefer a smaller immediate reward to a larger delayed reward, defined as impulsivity in opposition to self-control (Dixon et al., 2003). The hyperbolic equation *V* = *A*/(1+*kD*) was used to calculate the degree of delay discounting. In this equation, *V* is the subjective value of the delayed reward, *A* is the nominal amount of the delayed reward, *D* is the length of the delay, and *k* is a free parameter of delay discounting (i.e., discounting rate). A larger *k-*value describes a higher degree of delay discounting. In this study, we used an adapted version among Chinese students (Sun and Li, 2011). Examples for this version are “*A: receiving ¥9000 now; B: receiving ¥10000 one year later*” and “*A: receiving ¥1000 now; B: receiving ¥10000 one year later.*” Consistent with previous literature, *k*-values were calculated and log-transformed. The PDT is a three-part monetary-choice questionnaire with 10 questions in each part. Participants were instructed to circle their preferred outcome. One outcome was a smaller amount of money delivered “for sure” and the other was a larger amount of money delivered probabilistically (e.g., “$40 for sure” vs. “a 5-in-10 chance (50%) of winning $100”). The degree of probability discounting was calculated by the equation *V* = *A*/(1+*h*Θ), which uses the parameter Θ = (1-*p*)/*p* to substitute the odds against winning for the delay, describing hyperbolically declining subjective values of probabilistic outcomes (Rachlin et al., 1991). In this equation, the free parameter *h* refers to the degree of probability discounting. Lower *h* implie*s* that probabilistic rewards is less steeply discounted, suggesting a reduction in risk aversion. The PDT has been appropriately used among Chinese college students (Yan et al., 2016). In our study, the degree of probability discounting (*h*) is obtained and analyzed using the similar methods as in previous study (Madden et al., 2009). The *h* scores were also log-transformed to approximate a normal distribution.

### Statistical Analyses

Data were analyzed with the Statistical Package for the Social Sciences for Windows, Version 15.0 (SPSS Inc., Chicago, IL, United States). Chi-square tests were used to test between-group differences on categorical variables (i.e., gender, ethnicity, home locality, smoking, and drinking status), and *T*-tests were used to test group differences on age and Body Mass Index (BMI). MDMQ, DDT and PDT scores were compared between the groups using a 2 (group: binge-eating, non-binge-eating) × 2 (gender: male, female) multivariate analysis of variance (mANOVA) model. Partial correlations were tested between the MDMQ, DDT, PDT, and BES scores with age, gender, ethnicity, and home locality as the control variables. Complementally, a multivariable linear regression model was also conducted to test the effects of MDMQ, DDT and PDT scores on BES scores. Logistic regression was employed to examine the effects of MDMQ, DDT, and PDT scores on binge eating behavior, with gender as the control variable, given that the group difference on gender was significant. Multicollinearity was not a problem for any variable in theses regression models according to the variance inflation factor (VIF < 10). Statistical significance was set at *p* < 0.05, two-tailed.

## Results

### Group Differences on Demographics and Decision-Making Measures

**Table 1** describes the demographics and decision-making scores of the groups. In keeping with the literature (Dingemans et al., 2002), females were more likely than males to be involved in binge eating (χ^2^ = 20.547, *p* < 0.001), thus in further analyses gender was controlled as a between-group variable. No significant between-group differences were observed for age (*t* = -0.157, *p* = 0.875), ethnicity (χ^2^= 0.001, *p* = 0.998), home locality (χ^2^= 0.259, *p* = 0.611), BMI (*t* = 0.80, *p* = 0.424), smoking (χ^2^= 0.015, *p* = 0.902) or drinking (χ^2^= 1.750, *p* = 0.626) status.

**Table 1:** Demographic characteristics and scale scores of the sample (*N* = 1013).

Variables	Binge-Eating Group (*n* = 85)	Non-Binge-Eating Group (*n* = 928)	χ^2^*/t*	*p-*values
Age, years (*M* ± *SD*)	18.84 ± 0.86	18.85 ± 0.84	-0.157	0.875
Gender, Female *n* (%)	69 (81.2)	518 (55.8)	20.547	0.000
Ethnicity, Hans *n* (%)	50 (58.8)	546 (58.8)	0.001	0.998
Home locality, Urban *n* (%)	23 (27.1)	228 (24.6)	0.259	0.611
BMI, kg/m^2^ (*M* ± *SD*)	21.0 ± 2.58	20.71 ± 3.18	0.800	0.424
Smokers, *n* (%)	4 (4.7)	41 (4.4)	0.015	0.902
Drinking status, *n* (%)	Question: How many days you have at least one drink of alcohol during the past 30 days?			
Never	58 (68.2)	589 (63.5)		
1 or 2 days	5 (5.9)	55 (5.9)	1.750	0.626
3–9 days	1 (1.2)	33 (3.6)		
≥10 days	21 (24.7)	251 (27.0)		
BES score (*M* ± *SD*)	21.49 ± 3.49	7.28 ± 4.31	35.159	0.000
**MDMQ score (*M* ± *SD*)**				
Vigilance	6.98 ± 2.70	7.37 ± 2.64	-1.315	0.189
Procrastination	5.32 ± 2.40	3.93 ± 2.29	5.318	0.000
Buck-passing	5.84 ± 2.90	4.36 ± 2.82	4.604	0.000
Hypervigilance	5.29 ± 2.28	4.24 ± 2.15	4.311	0.000
**DDT score (*M* ± *SD*)**				
*k*/log-transformed	0.29 ± 0.22/-0.68 *±* 0.38	0.29 ± 0.20/-0.66 *±* 0.35	0.009/0.486	0.993/0.627
**PDT score (*M* ± *SD*)**				
Part A ($20 vs. $80):	5.67 ± 4.94/0.55 ± 0.48	5.20 ± 4.76/0.50 ± 0.48	0.868/0.946	0.386/0.344
*h*/log-transformed				
Part B ($40 vs. $100):	3.67 ± 4.06/0.34 ± 0.45	3.26 ± 4.11/0.25 ± 0.47	0.887/1.678	0.375/0.094
*h*/log-transformed				
Part C ($40 vs. $60):	3.09 ± 5.15/0.07 ± 0.54	2.26 ± 3.89/0.01 ± 0.47	1.834/1.043	0.067/0.297
*h*/log-transformed				

*BES, Binge Eating Scale; BMI, Body Mass Index; MDMQ, Melbourne Decision-Making Questionnaire; DDT, Delay-discounting Test; PDT, Probability Discounting Test, k represents the delay discounting rate, and h represents the probability discounting rate.*

On the MDMQ, the 2 (group: binge-eating, non-binge-eating) × 2 (gender: male, female) mANOVA model revealed significant group differences on Procrastination [*F*_(1,1009)_ = 13.338, *p* < 0.001, $\eta_{p}^{2}$ = 0.013], Buck-passing [*F*_(1,1009)_ = 6.141, *p* = 0.013, $\eta_{p}^{2}$ = 0.006], and Hypervigilance [*F*_(1,1009)_ = 4.403, *p* = 0.036, $\eta_{p}^{2}$ = 0.004] except Vigilance [*F*_(1,1009)_ = 3.035, *p* = 0.082]. Simple comparisons displayed that the binge-eating group had higher scores than the non-binge-eating group on Procrastination (*M*_*d*_ = 1.197, *p* < 0.001, Cohen’s *d* = 0.52), Buck-passing (*M*_*d*_ = 0.999, *p* = 0.013, Cohen’s *d* = 0.35), and Hypervigilance (*M*_*d*_ = 0.640, *p* = 0.036, Cohen’s *d* = 0.30). The mANOVA model also showed significant gender differences on Buck-passing [*F*_(1,1009)_ = 4.749, *p* = 0.030, $\eta_{p}^{2}$ = 0.005] and Hypervigilance [*F*_(1,1009)_ = 10.995, *p* = 0.001, $\eta_{p}^{2}$ = 0.011] but not on Vigilance [*F*_(1,1009)_ = 0.807, *p* = 0.369] or Procrastination [*F*_(1,1009)_ = 1.255, *p* = 0.263]. Simple comparisons found that females scored higher than males on Buck-passing (*M*_*d*_ = 0.878, *p* = 0.030, Cohen’s *d* = 0.31] and Hypervigilance (*M*_*d*_ = 1.011, *p* = 0.001, Cohen’s *d* = 0.47). There were no significant interaction effects of group **×** gender on Vigilance [*F*_(1,1009)_ = 1.611, *p* = 0.205], Procrastination [*F*_(1,1009)_ = 0.606, *p* = 0.436], Buck-passing [*F*_(1,1009)_ = 2.930, *p* = 0.087], or Hypervigilance [*F*_(1,1009)_ = 1.984, *p* = 0.159].

On the DDT, the 2 (group: binge-eating, non-binge-eating) × 2 (gender: male, female) mANOVA model did not find significant group differences on the *k-*values (log-transformed) [*F*_(1,1009)_ = 0.050, *p* = 0.823]. There were also no significant gender differences or interaction effects of group × gender on the *k-*values (log-transformed) [*F*_(1,1009)_ = 3.725, *p* = 0.054; *F*_(1,1009)_ = 0.109, *p* = 0.741, respectively]. On the PDT, the 2 (group: binge-eating, non-binge-eating) × 2 (gender: male, female) mANOVA model revealed no significant group differences on the *h-*values (log-transformed) of Part A ($20 vs. $80), Part B ($40 vs. $100), or Part C ($40 vs. $60) [*F*_(1,1009)_ = 0.002, *p* = 0.965; *F*_(1,1009)_ = 3.018, *p* = 0.083; *F*_(1,1009)_ = 0.495, *p* = 0.482, respectively]. There were also no significant gender differences on the *h-*values (log-transformed) of Part A, Part B, or Part C [*F*_(1,1009)_ = 1.966, *p* = 0.161; *F*_(1,1009)_ = 0.599, *p* = 0.439; *F*_(1,1009)_ = 0.006, *p* = 0.936, respectively]. The interaction effects of group × gender on the *h*-values (log-transformed) of the three parts were not significant [*F*_(1,1009)_ = 0.910, *p* = 0.340; *F*_(1,1009)_ = 0.317, *p* = 0.574; *F*_(1,1009)_ = 0.079, *p* = 0.779, respectively].

### Partial Correlations and Multivariable Linear Regression

**Table 2** displayed the partial correlations between decision-making measures and BES scores, with age, gender, ethnicity, and home locality as the control variables. Data revealed significant positive correlations between BES scores and MDMQ Procrastination, Buck-passing as well as Hypervigilance (*r*_*p*_ = 0.189–0.264, *p*s < 0.001). However, no significant associations were detected between BES scores and MDMQ Vigilance, DDT *k-*values (log-transformed), and PDT *h-*values (log-transformed) of Part A, Part B, and Part C (*p*s > 0.05).

**Table 2:** Partial correlations (*r*_*p*_) between decision-making measures and BES scores (*N* = 1013).

Variables	1	2	3	4	5	6	7	8	9
(1) BES score	-								
(2) MDMQ Vigilance	-0.051	-							
(3) MDMQ Procrastination	0.243***	0.115***	-						
(4) MDMQ Buck-passing	0.189***	-0.065	0.432***	-					
(5) MDMQ Hypervigilance	0.264***	0.187***	0.477***	0.458***	-				
(6) DDT *k* (log-transformed)	0.031	-0.029	0.005	0.010	0.028	-			
(7) PDT Part A *h* (log-transformed)	-0.011	-0.006	0.001	0.026	-0.037	-0.038	-		
(8) PDT Part B *h* (log-transformed)	0.001	-0.012	0.013	0.028	-0.031	-0.050	0.721***	-	
(9) PDT Part C *h* (log-transformed)	-0.014	0.014	0.026	0.014	-0.031	-0.038	0.464***	0.639***	-

*BES, Binge Eating Scale; MDMQ, Melbourne Decision-Making Questionnaire; DDT, Delay-discounting Test; PDT, Probability Discounting Test, k represents the delay discounting rate, and h represents the probability discounting rate. Control variables: age, gender, ethnicity, and home locality. ^∗∗∗^p < 0.001.*

A multivariable linear regression model was further used to test the effects of MDMQ, DDT, and PDT scores on BES scores with a 2-step design (i.e., gender was entered in step 1 as the control variable, and the decision-making variables were entered in step 2 as the predictors). **Table 3** displayed that MDMQ Procrastination, Buck-passing, and Hypervigilance were the positive predictors for BES scores after excluding the effects of gender [*F*_(9,1003)_ = 23.808, *p* < 0.001; Δ*R*^2^ = 0.092, *p* < 0.001].

**Table 3:** Multivariable linear regression analyses of decision-making measures on BES scores (*N* = 1013).

Models	Standardized Coefficients (β*)*	*t*	*F*	*R*	*R*^2^	*R*^2^ change
**Step 1**			92.906^∗∗∗^	0.290	0.084	0.084^∗∗∗^
Gender (Male = 1)	-0.290	-9.639^∗∗∗^				
**Step 2**			23.808^∗∗∗^	0.420	0.176	0.092^∗∗∗^
Gender (Male = 1)	-0.253	-8.608^∗∗∗^				
MDMQ Vigilance	-0.037	-1.089				
MDMQ Procrastination	0.141	4.149^∗∗∗^				
MDMQ Buck-passing	0.087	2.938^∗∗^				
MDMQ Hypervigilance	0.187	5.264^∗∗∗^				
DDT *k* (log-transformed)	0.030	1.030				
PDT Part A *h* (log-transformed)	-0.015	-0.352				
PDT Part B *h* (log-transformed)	0.026	0.552				
PDT Part C *h* (log-transformed)	-0.019	-0.517				

*BES, Binge Eating Scale; MDMQ, Melbourne Decision-Making Questionnaire; DDT, Delay-discounting Test; PDT, Probability Discounting Test, k represents the delay discounting rate, and h represents the probability discounting rate. Dependent variable: BES scores. ^∗∗^p < 0.01, ^∗∗∗^p < 0.001.*

### Logistic Regression Outcomes

A binary logistic regression model was conducted fundamentally to test the effects of decision-making measures on binge eating behavior comparing the two groups (i.e., binge eating vs. non-binge eating). A 2-step design was used: gender was entered in step 1 as the control variable, and the four MDMQ dimensions (Vigilance, Procrastination, Buck-passing, and Hypervigilance), DDT *k-*values (log-transformed) and PDT *h-*values (log-transformed) of Part A, Part B, and Part C were entered in step 2. **Table 4** revealed that only MDMQ Procrastination positively predicted binge eating (OR = 1.191, *p* < 0.01; Nagelkerke *R*^2^ = 0.130 for the model). However, none of the MDMQ Vigilance, Buck-passing and Hypervigilance, DDT *k-*values (log-transformed), and PDT *h-*values (log-transformed) of three parts displayed a significant predictive effect on binge eating behavior.

**Table 4:** Logistic regression analyses of decision-making scores on binge eating controlling for gender.

Models	Non-Binge Eating vs. Binge Eating^a^		
	(Binge Eating = 1)		
	B	Wald χ^2^	OR (95% CI)
**Step 1**			
Gender (Male = 1)	-1.228	18.525^∗∗∗^	0.293 (0.168–0.512)
**Step 2**			
MDMQ Vigilance	-0.076	3.021	0.927 (0.850–1.010)
MDMQ Procrastination	0.175	8.971^∗∗^	1.191 (1.062–1.335)
MDMQ Buck-passing	0.075	2.411	1.078 (0.980–1.185)
MDMQ Hypervigilance	0.080	1.404	1.083 (0.949–1.236)
DDT *k* (log-transformed)	-0.007	0.001	0.993 (0.519–1.898)
PDT Part A *h* (log-transformed)	-0.246	0.491	0.782 (0.393–1.555)
PDT Part B *h* (log-transformed)	0.629	2.103	1.875 (0.802–4.387)
PDT Part C *h* (log-transformed)	-0.109	0.114	0.897 (0.478–1.685)

*MDMQ, Melbourne Decision-Making Questionnaire; DDT, Delay-discounting Test; PDT, Probability Discounting Test, k represents the delay discounting rate, and h represents the probability discounting rate. CI, confidence interval; OR, odds ratio, ^a^N = 1013, Nagelkerke R^2^ = 0.130. ^∗∗^p < 0.01, ^∗∗∗^p < 0.001.*

## Discussion

This study compared different measures of decision-making in young adults with and without binge eating behavior. Our data revealed elevated scores on maladaptive decision-coping patterns of the MDMQ including Procrastination, Buck-passing, and Hypervigilance in the binge-eating group in comparison to the non-binge-eating group. Significant positive associations were found between BES scores and Procrastination, Buck-passing as well as Hypervigilance scores, and Procrastination, Buck-passing, and Hypervigilance were positive predictors of BES scores. More interesting, logistic regression model revealed that only Procrastination positively predicted binge eating. These findings support the hypothesis that diverse dimensions of decision-making are distinctly linked to binge eating and specific decision-making trait (i.e., Procrastination) may characterize individuals with binge eating, putatively representing an important vulnerability trait for the development of BED.

Decision-making deficit is considered an important neurocognitive characteristic of addictive behaviors (Redish et al., 2008; Leeman and Potenza, 2012; Yan et al., 2014), and poor decision-making might facilitate overeating since the remarkable clinical parallels between BED and addictions. Though several previous studies have investigated decision-making performance on cognitive tasks (mainly the IGT) among BED patients (Davis et al., 2010; Svaldi et al., 2010; Danner et al., 2012; Aloi et al., 2015), little work has paid close attention to other facets of decision making such as decision-coping traits and reward discounting. To our knowledge, this is the first study to contrast individuals with and without binge eating on a battery of decision-making measurements (i.e., decision-coping styles, delay discounting, and probability discounting). Our data demonstrated that non-clinical individuals with binge eating had more maladaptive decision-coping traits (i.e., Procrastination, Buck-passing, and Hypervigilance) than individuals without binge eating, suggesting a defective decision-making model at the personality level (Gorodetzky et al., 2011). This result is similar with the present findings of maladaptive decision-making styles in other addictions including stimulant and opiate addicts, problem drinking and gambling individuals as well as nicotine and caffeine dependents (Gorodetzky et al., 2011; Phillips and Ogeil, 2011; Phillips and Ogeil, 2015). It is noteworthy that in our study, though the binge-eating group did show higher scores on Buck-passing and Hypervigilance than the non-binge-eating group with medium to small effect sizes (Cohen’s *d =* 0.35, 0.30, respectively), and the partial correlations between Buck-passing, Hypervigilance and BES scores were significantly positive, Buck-passing and Hypervigilance did not display any main effects as a predictor on distinguishing binge eating from non-binge eating behaviors in the logistic regression model. This issue might be partly accounted for by the results that females scored significantly higher than males on both Buck-passing and Hypervigilance, and the proportion of females was significantly higher in the binge-eating group than in the non-binge-eating group (**Table 1**). In addition, the data of DDT and PDT did not reveal significant between-group differences, inconsistent with previous research data showing increased discounting of delayed rewards in women with obesity and BED compared to normal-weight women (Davis et al., 2010) and decreased discounting of probabilistic rewards in pathological gamblers compared with matched controls (Miedl et al., 2012), which might be due to the different methodologies and samples, therefore universal measurements should be adopted in further studies to clarify on the divergence of results. Furthermore, previous research has displayed diminished bilateral ventral striatal activity during monetary reward/loss processing in individuals with BED and obesity relative to non-BED individuals with obesity (Balodis et al., 2013a), suggesting that potential heterogeneity and neural differences of reward processing in these disorders should be taken into consideration in future.

More importantly, this study found that the binge-eating group scored higher on Procrastination than the non-binge-eating group with a medium to large effect size (Cohen’s *d* = 0.52), and only Procrastination positively predicted binge-eating behavior in the logistic regression model controlling for gender. Procrastination refers to a tendency of defensive avoidance that the decision maker escapes conflict by procrastinating decisions to bolster the least objectionable alternative, leading in turn to faulty decisions (Mann et al., 1997). Our study presented the first direct evidence in non-treatment seeking populations showing that specific trait of decision-making (Procrastination) is overtly increased in binge eating as a predictive indicator. The data, together with previous preliminary evidence in drug addiction (Gorodetzky et al., 2011; Phillips and Ogeil, 2011), suggest that Procrastination as a personality trait of decision-making probably characterizes individuals with BED. These findings support the hypothesis that Procrastination is a specific decision-coping trait involved in BED, putatively representing an important vulnerability for this disorder. Nevertheless, it remains unclear whether Procrastination predates BED or is a consequence of the pathology, considering the cross-sectional design of our study. Therefore, longitudinal designs should be adopted in future studies. Moreover, in consideration of the clinical similarity between BED and substance use disorders, the current findings also call into future studies on the potential neurobiological mechanisms of Procrastination in both disorders. Especially, individuals with BED and obesity were partly differentiated by hypoactivity in brain areas involved in inhibitory control (i.e., ventromedial prefrontal cortex, inferior frontal gyrus, and insula) compared to non-BED individuals with obesity and lean comparison participants (Balodis et al., 2013b). Thus further studies employing neuroimaging methods (e.g., functional magnetic resonance imaging, fMRI) should be of help to elucidate the neural basis of Procrastination in BED.

There were several limitations in this study. First of all, the cross-sectional study design was not able to determine causal relationships between the decision-making measures and binge eating behavior. Although the data suggest that Procrastination might increase risk for BED, further longitudinal studies are warranted. Secondly, BED conditions and decision-making dimensions were evaluated by self-report questionnaires, which could be liable to bring bias into the data analyses. Thus, the results should be explained carefully. Thirdly, the participants consisted of university students, and especially, current and lifetime psychiatric and mental disorders that have been exhibited to be mostly comorbid with BED were excluded in this study (primarily for the purpose of directly portraying a “pure” decision-making patterns in binge-eating behavior itself), so the findings cannot be generalized to the whole population of BED, and the differences on decision-making models between different BED samples (e.g., college students, community populations, clinical patients) should be examined in future research. Besides, the current findings mainly focused on the decision-coping aspects in binge eating, but actually, other cognitive mechanisms such as planning abilities and cue-induced risky decision-making could also play an important role underlying BED (Neveu et al., 2014, 2016), which should be encompassed more comprehensively in future studies.

Despite these limitations, our results indicate that Procrastination, Buck-passing, and Hypervigilance are increased among individuals with binge eating compared to those without binge eating, and moreover, Procrastination is a risk factor in predicting binge eating behavior, putatively identified as a vulnerability trait of BED. The findings may be conducive to further absorbing the mechanisms of specific decision-making traits implicated in the development of BED, and facilitating the exploitation of effective prevention and early interventions of compulsive overeating.

## Ethics Statement

The procedures reported in this study were reviewed and approved by the Human Research Ethics Committee at the Guizhou Medical University, and the proposed recruitment process, study design, and plans to compensate participants were carried out in accordance with the Declaration of Helsinki.

## Author Contributions

W-SY designed the study, wrote the protocols, directed the study, and wrote the first draft of the manuscript. R-RZ performed the assessments and data collection. YL and Z-ML assisted with main data analysis. Y-HL as well as the other authors contributed to the writing and all authors approved the final manuscript.

## Conflict of Interest Statement

The authors declare that the research was conducted in the absence of any commercial or financial relationships that could be construed as a potential conflict of interest.
